# Canine models of spine disorders

**DOI:** 10.1002/jsp2.1109

**Published:** 2020-07-20

**Authors:** Naomi N. Lee, Jacob S. Kramer, Aaron M. Stoker, Chantelle C. Bozynski, Cristi R. Cook, James T. Stannard, Theodore J. Choma, James L. Cook

**Affiliations:** ^1^ Department of Orthopaedic Surgery University of Missouri Columbia Missouri USA; ^2^ Thompson Laboratory for Regenerative Orthopaedics University of Missouri Columbia Missouri USA; ^3^ Comparative Medicine Program University of Missouri Columbia Missouri USA

**Keywords:** canine research models, intervertebral disc degeneration, spine pathology, spine‐related disorders

## Abstract

Neck and low back pain are common among the adult human population and impose large social and economic burdens on health care and quality of life. Spine‐related disorders are also significant health concerns for canine companions with etiopathogeneses, clinical presentations, and diagnostic and therapeutic options that are very similar to their human counterparts. Historically, induced and spontaneous pathology in laboratory rodents, dogs, sheep, goats, pigs, and nonhuman primates have been used for study of human spine disorders. While each of these can serve as useful preclinical models, they all have inherent limitations. Spontaneously occurring spine disorders in dogs provide highly translatable data that overcome many of the limitations of other models and have the added benefit of contributing to veterinary healthcare as well. For this scoping review, peer‐reviewed manuscripts were selected from PubMed and Google Scholar searches using keywords: “intervertebral disc,” “intervertebral disc degeneration,” “biomarkers,” “histopathology,” “canine,” and “mechanism.” Additional keywords such as “injury,” “induced model,” and “nucleus degeneration” were used to further narrow inclusion. The objectives of this review were to (a) outline similarities in key features of spine disorders between dogs and humans; (b) describe relevant canine models; and (c) highlight the applicability of these models for advancing translational research and clinical application for mechanisms of disease, diagnosis, prognosis, prevention, and treatment, with a focus on intervertebral disc degeneration. Best current evidence suggests that dogs share important anatomical, physiological, histological, and molecular components of spinal disorders in humans, such that induced and spontaneous canine models can be very effective for translational research. Taken together, the peer‐reviewed literature supports numerous advantages for use of canine models for study of disorders of the spine when the potential limitations and challenges are addressed.

## Graphical abstract

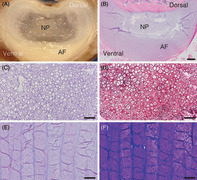

## 1 INTRODUCTION

Disorders of the spine comprise a major global healthcare concern in terms of pain, disability, and associated costs. While tremendous efforts and funding have been poured into spine research, and advances in basic and clinical science have been realized, translational animal models that effectively bridge the gap between bench and bedside appear to be underused. Induced and spontaneous canine models can be very effective in providing preclinical evidence to address this unmet need. Importantly, disorders of the spine affect canine patients with similar prevalence and impact to that seen in human patients, such that translational potential is high, and results can be applied to clinical veterinary medicine as well. Therefore, the objectives of the present review are to outline the applicable similarities in the key features of spine disorders between dogs and humans, describe relevant canine models, and highlight the applicability of these models for advancing understanding in mechanisms of disease, diagnosis, prognosis, prevention, and treatment of spine pathology.

## 2 INTERVERTEBRAL DISC DISEASE IN HUMANS AND DOGS

In humans, symptomatic disorders of the spine are typically classified into one of four categories: axial back/neck pain syndromes, stenosis syndromes, instabilities, and deformities. While there are distinct features of each, there is also considerable overlap. Axial pain syndromes have had several distinct etiologies implicated to include paraspinal muscle dysfunction,^1^ facet joint arthrosis,^2^ inflammatory arthritides (including enthesitis),^3^ and intervertebral disc degeneration (IVDD).^4^

Stenosis syndromes in humans typically take the form of compressive myelopathies, postural dysfunction of the cauda equina (“neurogenic claudication”), or focal radiculopathies resulting from acute disc herniation or impingement from osteophytes.^5^ More acute compressive pathologies are typically due to traumatic injury, infection, or tumor. These disorders can generate a very wide range of clinical symptoms, from episodic, mildly bothersome paresthesias and aches to very debilitating nervous system dysfunction as in paraplegia and quadriplegia.

Instabilities likewise can have a variety of etiologies, from acute traumatic fractures and/or dislocations to pathologic destruction due to tumor or infection^6^ to gradual degenerative spondylolisthesis or cranial settling as seen in inflammatory arthritides. All instabilities result from loss of normal musculoskeletal restrains on spinal segment motion that then allow for segmental motions that could be injurious to the contained neurological structures.

Deformities represent deviations in the normal three‐dimensional shape of the spine such as scoliosis or hyperkyphosis. In the most serious cases, deformities lead to an imbalance of the spine in which the patient's head is no longer centered over the sacrum, making ambulation much less energy efficient and causing significant disability. Potential causes for deformities are developmental, degenerative, or following destruction due to trauma, tumor, or infection.^7^

A similar spectrum of pathology has been reported for canine patients with similar categorization algorithms. Categorization may also be based on the anatomic structure(s) considered to be the primary source of pathology and/or pain generators, including disc, endplate, facet joint, and muscle‐tendon (Table 1).^8^ While disc‐driven IVDD is far and away the most prevalent and most studied, endplate‐driven, facet‐driven, and muscle‐driven disorders of the spine have been reported as well. Endplate‐driven disorders in dogs include discospondylitis, fatty infiltration, dysplasia/remodeling, osteochondrosis, and Schmorl's nodes. Based on diagnostic imaging studies,^9^, ^10^ the lumbosacral (LS;L7‐S1 in dogs) region has a predilection for endplate pathology. Fatty infiltration of endplates predominantly occurs in small breed dogs, especially chondrodystrophic (CD) breeds, and may be found anywhere along the spine. All other types of endplate lesions are more prevalent in medium and large breed dogs with discospondylitis being most common followed by sclerotic/reactive/degenerative changes, osteochondrosis, and Schmorl's nodes.^9^ Endplate dysplasia, sclerosis, remodeling, and/or degeneration are associated with vertebral instability in the canine LS region (LS instability) and caudal cervical region (caudal cervical spondylomyelopathy [CCSM] or Wobblers syndrome), both of which typically include some degree of IVDD.^11^, ^12^, ^13^ CCSM is most common in Great Danes and Doberman Pinschers, while LS instability occurs most frequently in German Shepherd Dogs, Border Collies, Australian Shepherds, Labrador Retrievers, Rottweilers, Bernese Mountain Dogs, Boxers, Dalmatians, and Irish Setters. LS instability appears to have genetic^15^, ^16^ and biomechanical (activity‐related)^17^, ^18^, ^19^, ^20^, ^21^, ^22^, ^23^, ^24^, ^25^, ^26^ components, and is being diagnosed more commonly in dogs with the growth in number of working, service, and performance dogs worldwide, as well as availability and use of advanced diagnostic imaging in veterinary medicine.^12^, ^26^, ^27^, ^29^, ^30^, ^31^, ^32^ Many dogs affected with LS instability have larger, less sagittally oriented facet joints at L7‐S1, which are associated with increased LS flexion and extension,^33^ and both LS instability and CCSM can also be considered in the facet‐driven category based on concurrent dysplasia, remodeling, and degeneration of affected facet joints.^11^, ^12^, ^13^, ^15^, ^16^ Other facet‐driven disorders in dogs include hypoplasia/aplasia^34^ and osteoarthritis.^35^ In terms of muscle‐driven disorders of the canine spine, muscular dystrophy in Golden Retrievers^36^ spinal muscular atrophy in Brittany Spaniels^37^ have been reported. Spondylosis deformans, diffuse idiopathic skeletal hyperostosis (DISH), and scoliosis may also involve muscle‐driven mechanisms.

**TABLE 1:** Canine models and disorders

Category	Canine disorders
Disc^8^, ^38^, ^39^, ^40^, ^41^, ^42^, ^43^, ^44^, ^45^, ^46^, ^47^, ^85^, ^86^, ^87^, ^88^, ^89^, ^91^, ^92^, ^93^, ^94^, ^95^, ^96^, ^97^, ^98^	Intervertebral disc disease (Hansen type 1 and 2)
Endplate^8^, ^9^, ^10^, ^11^, ^12^, ^13^, ^15^, ^16^, ^17^, ^18^, ^19^, ^20^, ^21^, ^22^, ^23^, ^24^, ^25^, ^26^, ^27^, ^28^, ^29^, ^30^, ^31^, ^32^	Discospondylitis Fatty infiltration Dysplasia Remodeling Osteochondrosis Schmorl's nodes Wobblers (CCSM) Lumbosacral instability
Facet joint^8^, ^11^, ^12^, ^13^, ^15^, ^16^, ^33^, ^34^, ^35^	Hypoplasia Aplasia Osteoarthritis Wobblers (CCSM) Lumbosacral instability
Muscle‐tendon^8^, ^34^, ^35^, ^36^	Muscular dystrophy Spinal muscular atrophy Spondylosis deformans DISH Scoliosis

It is likely that there is a large degree of crossover with respect to the anatomic “drivers” of spine disorders in both canine and human patients. As such, it is important to consider the whole organ or functional spinal unit (FSU) and whole body when treating patients and modeling disease. Since the majority of clinical disease and related research have centered on the intervertebral disc (IVD) and endplate‐driven, facet‐driven, and muscle‐driven disorders typical involved or affect the disc, the present review focuses on IVD disease and degeneration.

IVDD is often characterized by loss of water from the nucleus pulposus (NP) with associated alterations in disc composition and structure, reducing its ability to function as a hydraulic cushion in vertebral column loading bearing and motion.^38^, ^39^, ^40^ As degeneration progresses in dogs and humans, NP cells form large clusters and shift from collagen II to collagen I synthesis further compromising the critical biomechanical balance that determines its material properties and function.^41^, ^42^ Annulus fibrosus (AF) cells and matrix also undergo degenerative changes in unstable or weak regions of the disc. There is evidence that inflammatory and degradative processes drive IVDD in both dogs and humans.^42^, ^43^, ^44^, ^45^ As IVDD progresses, significant changes in articular facets, vertebral endplates and bodies, ligaments, and musculature ensue.^46^, ^47^ As with any animal model, there are associated limitations that should be considered when using and translating data from canine studies toward understanding human disease. Anatomically, the canine has seven lumbar IVDs while the human only has five. Table 2 shows comparative vertebral anatomy between human and canine spines. Structurally, canine IVDs have a secondary center of ossification that is not present in human IVDs. However, clinical, compositional, histologic, and biomechanical similarities between canine and human IVDD have allowed for comparative research to evaluate aspects of degeneration that may translate to either species making canine models of IVDD extremely useful research tools.

**TABLE 2:** Comparative anatomy of canine and human vertebral columns

	Human	Canine (NCD)	Canine (CD)
Vertebral formula	C7; T12; L5; S5 (fused)	C7; T13; L7; S3; Cd variable	
Most commonly affected IVD levels in clinical patients	C5‐C7 L4‐S1	C5‐T1 L6‐S1	C2‐C3 T12‐L1

## 3 EX VIVO MODELS

Models using cells, single tissues, or whole organs provide a controlled method for investigating mechanisms of disc degeneration.^48^ Cell culture models allow for control of certain variables and are typically less complex and expensive to employ than other options.^49^ However, extracellular matrix (ECM) is altered or absent, which commonly results in cell dedifferentiation and does not allow for valid assessment of biomechanics or morphological integrity.^50^ Tissue cultures of IVDs without adjacent endplates allow for better maintenance of cell distribution and differentiation, ECM integrity, and material properties, but biologic and biomechanical influences of endplate cartilage and vertebral bone are lost, and the NP is allowed to freely swell in culture.^51^, ^52^, ^53^ Based on these limitations, whole organ IVD explant models have been developed in several species and used to study biologic and biomechanical components of the FSU in health and disease. Canine ex vivo models have been used effectively to address questions regarding nutrient and oxygen supply, osmolarity, cell phenotype, gene expression, cell signaling pathways, and biomarkers for diagnosis, staging, and therapeutic targets.^29^, ^54^, ^55^, ^56^, ^57^ Used in these ways, these models can serve as excellent screening tools for focused, efficient, and ethical use of animal models for translational studies toward clinical application.

## 4 CANINE MODELS

Numerous animal models have been developed to investigate specific questions about IVDD across the spectrum of disease mechanisms, diagnosis, staging, prevention, treatment, and prognostication. Animal models range from rodents to primates, induced to spontaneous, and acute to chronic with spontaneous disc degeneration in nonhuman primates, age‐related disc degeneration in mice, and genetically‐engineered spontaneous disc degeneration in mice having attractive modeling characteristics. When considering all of the factors involved in selecting an animal model including availability, ethics, cost, and translational applicability, canine models can also be considered strong candidates.^58^, ^59^, ^60^ Spontaneous and induced canine IVDD models have been used to investigate a wide spectrum of biologic, biomechanical, and clinical components of spine disorders in their human counterparts (Table 3).

**TABLE 3:** Induced, spontaneous, and combinations of IVDD canine models

Category	Method	Time points	Pathology	Intended application
Induced	Annular injury^66^	Baseline, 1 wk, 12 wks	Disc bulge or herniation	To determine the effects of annular injuries on response to mechanical demands measured by MRI
	Discectomy^58^	Baseline, 16 wks	Disc collapse, dehydration	To determine the radiographic and histological effects of discectomy procedures
	Endplate injury^65^	31‐70 wks following surgery	Degenerative histological changes	To determine the histological and biochemical effects of blocking endplate nutrition
	Chemical induction^70^	7 d‐3 y following surgery	Disc displacement, macroscopic degeneration	To determine the efficacy of chemonucleolysis in treating canine disc herniation
Spontaneous	Prospective, observational^45^	Canines <1 y old	Spontaneous disc degeneration	To determine the prevalence and severity of disc degeneration in canines
	Characterization, observational^79^	Animals grouped by increasing age	Naturally occurring degeneration	To determine the relationship between disc degeneration and age in beagle spine
Combinations	Therapy evaluation^67^	Baseline, 12 wks, 3, 6, 9, 12 mos	Induced degeneration treated with cell transplantation	To determine the if autologous disc derived cells can aid in healing a damaged disc
	Combination therapy^80^	7, 14, 30, 90, 150 d	Spontaneous disc herniation thoracolumbar	To determine the effect of physiotherapy on surgery outcome

### 4.1 Induced models

Induced models provide a method for creating standardized pathology to consistently initiate desired disease processes while mitigating confounding variables and associated variability. The primary types of induced models in dogs include surgical or chemical focal annular injury, removal of disc material, or a combination of these insults.

Annular injury is the most common induced IVDD model across species, having been used for nearly a century to consistently initiate degeneration of IVDs in dogs.^61^, ^62^ Annular injuries are induced by incision, puncture, or direct disruption of the AF and/or its attachment to the endplate. These models are intended to mimic IVDD resulting from annular tears in humans by introducing a small AF injury that leads to the known sequelae that result in symptomatic disc disease. These sequelae include structural compromise of the annulus, loss of resistance to hydrostatic forces within the disc, abnormal loading, apoptosis, necrosis, and cell phenotype shifts, NP protrusion/extrusion, extradiscal exposure of NP causing impingement and/or inflammatory responses, loss and remodeling of ECM, and ultimately, IVD failure.^63^ As such, annular injury models can allow for assessments of biochemical, histologic, and biomechanical perturbations that lead to the clinical manifestations of symptomatic IVDD. The primary limitations involve artificial ways in which the pathology is created, the relative severity of the injury and resultant timing and progression of disease, and the otherwise‐normal condition of the spine in the research dogs.

In an attempt to address these limitations, endplate models,^64^, ^65^ biomechanical injury models,^66^ and discectomy models^45^, ^58^ have been developed and implemented in dogs. Endplate models employ a mechanical disruption or physical barrier at the cartilaginous endplate with the goal of inhibiting IVD imbibition. The resulting endplate perfusion perturbations are thought to cause nutritional deficits in the disc, inducing degeneration. Initial data from this model showed ECM alterations and histopathology consistent with some components of degenerative disc disease in people.^64^

A biomechanical‐induced IVDD model has also been attempted in dogs by attaching coil springs to vertebral bodies to facilitate compressive overloading of discs.^66^ However, the investigators reported no macroscopic or radiographic indications of degeneration and only minimal histologic changes, suggesting that this biomechanical method may not have translational validity and highlighting the difficulty of using biomechanical insults in vivo.

In order to induce more expedient and severe inflammatory and degradative changes, surgically and chemically induced partial discectomy models have been employed in dogs. Surgical excision of a portion of the disc (subtotal discectomy) is the most common means of creating this model and has been used to study mechanisms and timing for disease processes, correlations among diagnostic and staging modalities, and safety and efficacy of potential therapies.^58^, ^67^, ^68^ Recently, percutaneous laser discectomies have become more widely performed to create discectomy models in order to avoid confounding variables associated with more invasive surgical models and be more directly translational in nature.^59^, ^60^, ^63^ However, direct comparisons among these models have not been reported to the authors' knowledge.

Chemically induced discectomy, or chemonucleolysis, uses enzymes to degrade the NP, effectively reducing its viability, volume, and material properties.^69^ Agents commonly used for IVD chemonucleolysis include chondroitinase or papain.^70^ To initiate disc degeneration, chemonucleolysis is dose‐dependent such that chemically induced models frequently require high concentrations of these agents to effectively result in relevant degenerative changes, which may limit translational potential.^71^ The benefits of chemically induced models include their capabilities for targeted damage without associated fibrosis and other confounding variables associated with surgically induced models.^72^ These models may also have therapeutic relevance in that chemonucleolysis has been successfully used as a treatment option in select human and veterinary patients.^69^, ^70^, ^72^, ^73^, ^74^

Surgically and chemically induced models have also been used in CD breeds of dogs in order to include the spontaneous‐disease components to methods for initiating and perpetuating insults.^45^, ^47^, ^75^, ^76^

### 4.2 Spontaneous models

In dogs, selective breeding has resulted in wide phenotypic diversity varying from teacup Yorkshire Terrier to Great Dane.^77^ Selective breeding and artificial selection created breeds with varying characteristics that have resulted in disorders that are similar to those in humans. As such, dogs are often used as valid translational models of human disorders.^78^ IVDD is a prime example of a disorder that is shared between the two species and for which dogs can serve as robust preclinical animal models.

Dog breeds can be categorized as either CD or nonchondrodystrophic (NCD) dogs with IVDD affecting both categories in different ways. The lifetime prevalence of IVDD in dogs has been estimated at between 2% and 5%^38^, ^39^, ^40^ with highest prevalence in in older dogs and in CD breeds such as Dachshund, Cocker Spaniels, and Beagle.^27^, ^39^, ^79^, ^80^ In Dachshunds, relative risk for IVDD is 10 to 12 times higher than other breeds with between 19% and 24% of Dachshunds showing clinical signs of IVDD during their lifetimes.^28^ Other CD breeds at higher risk for IVDD include Beagle, Cocker Spaniel, Cavalier King Charles Spaniel, Tibetan Spaniel, and Shih Tzu, while high‐risk NCD breeds include Doberman Pinscher, Papillon, Rottweiler, Dalmatian, German Shepherd Dog, Miniature Schnauzer, and Bernese Mountain.^27^ Furthermore, IVDD has been reported to be more pervasive in the purebred population compared to mixed‐breed dogs.^40^, ^81^

Spontaneous canine models of IVDD rely on naturally occurring degeneration to closely mimic the cumulative structural and metabolic changes that occur in association with human IVDD. Large population studies have estimated that between 71% and 77% of humans harbor degenerative discs before 50 years of age.^82^ Spontaneous or naturally occurring IVD degeneration appears to progress in similar ways clinically, macroscopically, histologically, and biochemically for both species,^45^ and disc herniation occurs at similar rates (approximately 2%) in humans and dogs.^39^, ^83^

In dogs, IVDD is categorized as Hansen Type I (calcified NP extrusion out of IVD through AF) or Hansen Type II (weakened AF protrudes outward into the vertebral canal).^27^, ^29^, ^30^, ^31^, ^45^, ^84^ CD breed dogs mostly present with Hansen Type I and NCD breed dogs often present with Hansen Type II. CD dogs encompass smaller breeds that are known to experience IVD degeneration at an earlier age than their NCD counterparts.^29^ Based on the earlier onset and typical clinical presentation, CD dogs, most often Beagles and Dachshunds, are used for spontaneous models of acute traumatic or overuse IVDD in younger patients,^29^, ^45^ whereas NCD dogs more closely model chronic IVDD in older patients. While disc degeneration and herniation are diagnosed less commonly in NCD dogs compared to CD dogs, degenerative changes do occur commonly in NCD dogs on a histologic level.^31^ Importantly, CD and NCD dogs have many characteristics that are similar to various clinical manifestations seen in human IVDD patients. Clinical signs, imaging findings, histology, treatments, biomechanics, and molecular markers of IVDD between the two species share numerous similarities. As such, degenerative and healthy discs from CD and NCD dogs can be assessed over the lifespan to study changes in cell phenotype (notochordal cells, NP cells), gene expression, signaling pathways, and ECM alterations in degenerative IVDs in order to better understand disease mechanisms, develop and validate biomarkers, and advance early diagnosis, prevention, treatment, and development of prognostic indicators for IVDD in dogs and humans.^29^, ^55^, ^56^

## 5 MECHANISMS OF IVDD

Based on shared features of development and progression, spontaneous IVDD in dogs is a powerful model to study mechanisms of disease for human IVDD.^45^, ^85^, ^86^, ^87^, ^88^ Recognized biologic mechanisms of IVD degeneration in both species include calcification of cartilage end plates reducing nutrient supply to the NP, increased cell death,^32^, ^87^, ^89^ loss of the notochordal cell population, and replacement with chondrocyte‐like cells of the NP,^85^ transition of the gel‐like NP to a more fibrous and/or chondroid^91^ tissue weakening of the AF through degeneration of the ECM and development of fissures and cracks,^86^ increased intrinsic and extrinsic tissue inflammation,^92^ and increased degradative enzyme production and activity.^43^, ^93^ However, the precise roles, interactions, links, and correlations among these mechanistic components of disease and their contributions to the various forms of symptomatic IVDD have not been fully characterized.

The IVDs of CD dogs undergo many of the changes that occur in human IVDs at an early age.^86^ Calcification of cartilage endplates and NP can occur as early as 5 months of age in CD dogs, and is observed in 31.2% of cervical and 43% of lumbar discs by 1 year of age.^86^ Relative within‐animal differences in degree and timing of calcification and associated pathology can be used to characterize drivers of IVD calcification and related clinical disease while reducing the number of animals needed for valid study.

In the NP, the notochordal cell population is lost in humans and CD dogs and replaced with a chondrocyte‐like cell population. This transition in cell population is associated with a shift in biochemical composition of the NP from a gel‐like tissue with a high proteoglycan‐to‐collagen ratio to a more fibrous and/or chondroid tissue with reduced proteoglycan and water content. Calcification of the cartilage endplates and a resultant reduction in nutrient delivery to and waste removal from the NP is believed to be a primary contributor to these degenerative changes in the NP. In NCD dogs, these changes in the NP occur less consistently and later in life compared to CD dogs. Therefore, comparative studies that use CD and NCD dogs can be designed to elucidate factors driving the age‐ and disease‐related changes that occur in the NP of dogs and humans.

Another key mechanism in IVD degeneration is cell death due to apoptosis and autophagy.^90^ The cells of the NP and AF are required to maintain the complex ECM of the IVD. Progressive loss of NP and AF cell content is a common feature with age and degeneration in both human and canine patients. Loss of cells is associated with ECM alterations and decreased integrity of both tissues, however, the order and sequence of events in this degenerative pathway and its effects on likelihood and timing of disc herniation are unknown. Herniation of the IVD occurs by one of two general mechanisms in human patient cohorts as well as in CD vs NCD dogs.^86^ Complete extrusion of the NP through the AF is common in traumatic disc ruptures in relatively younger human patients and is the Hansen type I herniation most commonly seen in CD dogs. IVD protrusion is most commonly noted in association with aging and/or chronic degenerative disc disease noted in relatively older human patients and NCD dogs. Spontaneous IVDD in CD and NCD dogs can provide novel insight into cell loss and matrix alterations in AF vs NP in contributing to distinct pathways for IVD herniation.

Inflammation and degradative enzyme activity have been observed in degenerative IVDs in human and canine patients.^32^, ^43^, ^92^, ^93^ While the inflammatory cytokines IL‐1β and TNF‐α have been implicated in increased cell death, increased degradative enzyme production, and decreased production of ECM proteins, the dynamics of cytokine involvement with age and degeneration are still incompletely characterized. However, the precise roles and effects of inflammatory cytokines and the dynamics of related matrix metalloproteinase, aggrecanase, and TIMP production in normal and degenerative IVDs tissues are not well delineated. Because CD and NCD dogs develop IVD degeneration at different rates and often at different ages, these spontaneous IVDD models can provide clinically relevant information on the roles of inflammatory and degradative mediators in acute and chronic degenerative disc diseases.

**FIGURE 1:**
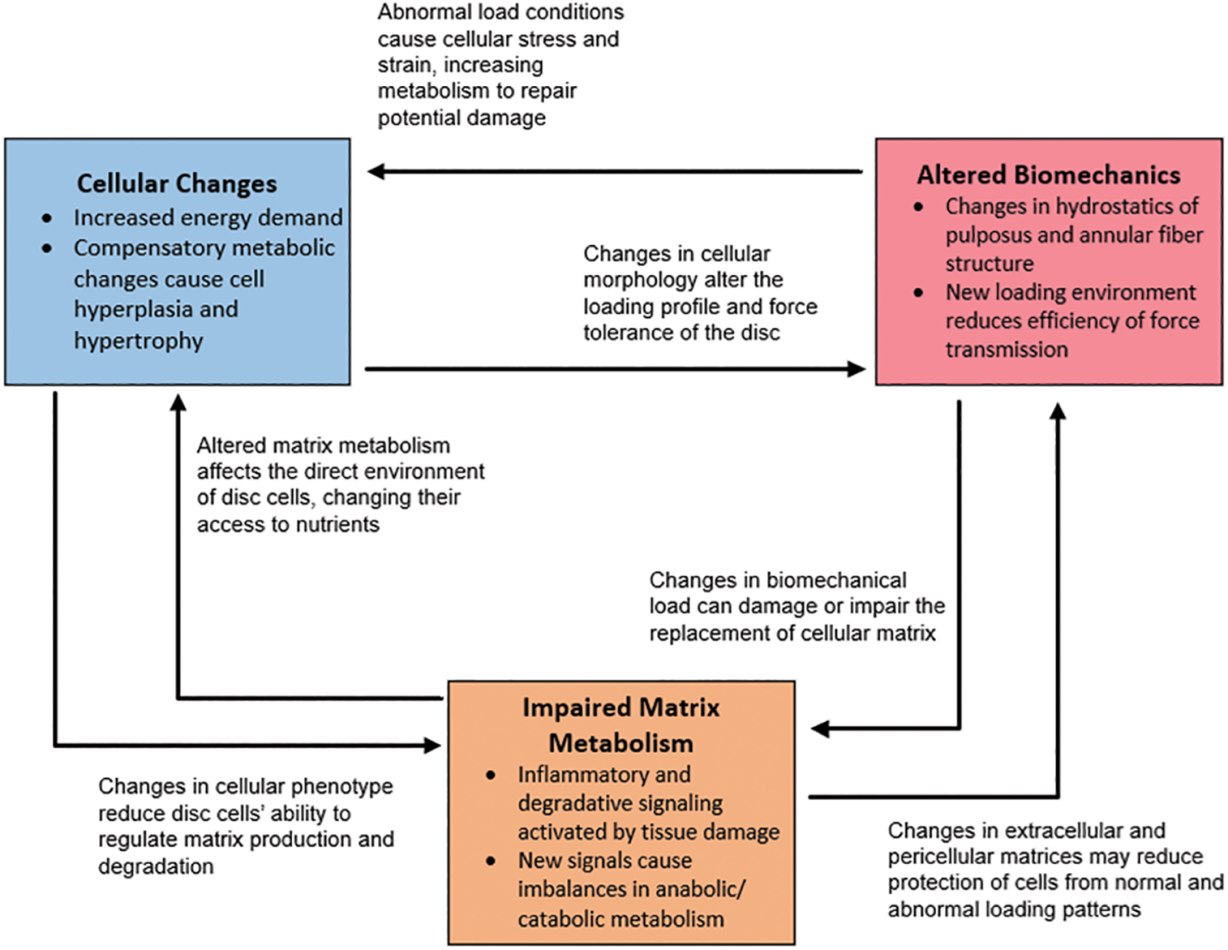
Biomechanical‐Biology of IVD cycle. Schematic depicting the main and peripheral features of cellular changes, impaired matrix metabolism, and altered biomechanics and their role in the degenerating intervertebral disc

While the biologic and biomechanical components of the spine are inextricably linked in IVD health and disease, they are often approached in separate, parallel pathways with respect to experimental design, outcome measures, and application. Primary biomechanical mechanisms for IVDD include deficiencies or failures to maintain hydrostatic pressure transduction, to transmit load, and/or to allow functional movements. For each of these disease mechanisms to be avoided, the composition, structure, and integrity of all components of the FSU including the NP, AF, endplates, vertebral bodies, facet joints, ligaments, and paraspinal muscles and tendons must be maintained in balance, relationship, and function.^91^ In the IVD, alterations in the critical balance of water, proteoglycan, and collagen composition and structure of the NP can cause rapid and profound loss of material properties that govern compressive load distribution,^94^ nutrient and waste transport,^95^ cell signaling, and mechanotransduction.^55^, ^95^ Similarly, physical and/or biochemical disruptions of the concentric rings of the AF and/or its attachments to the endplates negatively affect its ability to contain the NP and to effectively resist the omnidirectional hydrostatic pressures, load transmission, and stability requirements for posture and activity. These alterations and disruptions directly affect nutrient and waste diffusion, loading and movement of the FSU, and disc integrity.^94^, ^95^ Because the IVD relies on these biomechanical processes to maintain its health and function, loss of these inevitably propagates a vicious cycle of compensatory tissue remodeling, inflammation, degradation, dysmetabolism, degeneration, pain, and dysfunction.^91^, ^94^, ^95^, ^96^, ^97^ (Figure 1).

## 6 CAUSES OF IVDD IN DOGS

IVDD in humans and dogs is considered to be a complex multifactorial spectrum of disease influenced by genetics, aging, overuse, and/or trauma. Specific genes have been implicated in both species and many types of IVDD are considered familial.^1^, ^13^, ^15^, ^16^, ^17^, ^18^, ^19^, ^20^, ^21^, ^22^, ^23^, ^24^, ^25^, ^35^, ^41^, ^98^, ^99^, ^100^ Aging has significant effects on canine IVDs with strong evidence for progressive degenerative changes in discs and associated increased likelihood for symptomatic IVDD in older dogs.^27^, ^30^, ^45^, ^86^, ^101^, ^102^ Facet joints also have significant alterations with increasing age,^15^, ^33^ which further drive disc degeneration and associated morbidities.^47^ Environmental and lifestyle factors that increase biomechanical loading of IVDs, especially repetitively, are associated with IVDD and are more pronounced with increasing age.^22^, ^103^ While NCD dogs are relatively protected against IVDD in general, the incidence of IVDD increases in performance and working NCD dogs consistently experiencing repetitive movements of the spine under load.^17^, ^18^, ^19^, ^20^, ^21^, ^22^, ^23^, ^24^, ^25^, ^26^, ^45^, ^104^, ^105^ Overt trauma to the spine can also occur in these working and performance dogs, and a traumatic event (eg, jumping off the couch) is often reported in association with acutely symptomatic IVDD in CD dogs. Anatomical and biomechanical factors including spinal canal diameter, associated ligaments, epaxial and hypaxial musculature, flexion, extension, rotation, and loading moments on the spine likely influence overuse and traumatic causes of IVDD as well.

While obesity is accepted as a significant risk factor for symptomatic disc disease in humans,^106^, ^107^, ^108^, ^109^, ^110^, ^111^, ^112^ this association is less clear in canine patients. In general, obesity is considered a relative risk factor for IVDD in dogs,^79^, ^113^ however, in CD breeds, specifically Dachshunds, body condition score has not been reported to have a strong correlation with prevalence of IVDD.^114^, ^115^ This may be a true lack of higher risk or it may be that other risk factors for IVDD—such as disc calcification and spine biomechanics—predominate in CD dogs.

To the authors' knowledge, there are no data reporting the effects of cigarette smoking (second‐hand smoke) on IVDD in dogs. However, other animal models report that exposure to components of tobacco is associated with decreased nutrient transport, altered cell morphology and function, increased oxidative stress and cell death, decreased ECM content and synthesis, and structural changes in IVDs.^116^, ^117^, ^118^, ^119^, ^120^, ^121^, ^122^, ^123^, ^124^, ^125^, ^126^, ^127^ Tobacco use is clearly implicated in symptomatic disc disease in human patients.^110^, ^111^, ^128^ As such, research aimed at the effects of second‐hand smoke on canine companions could provide important insight into mechanisms for IVDD associated with tobacco use, as well as disc degeneration pathways, in general.

Diabetes mellitus (DM) is a chronic metabolic disorder that has been indicated as a risk factor for accelerating IVD degeneration in human patients.^128^, ^129^, ^130^, ^131^, ^132^, ^133^, ^134^, ^135^, ^136^, ^137^ DM is thought to accelerate IVD degeneration by increasing advanced glycation end‐product (AGE) accumulation in discs.^138^, ^139^, ^140^, ^141^, ^142^ Studies examining the degenerative effects of AGEs on IVDs have been performed in murine models primarily. Dogs are affected by DM and require monitoring and insulin therapies such that diabetic dogs could serve as a valid large animal model for study of DM‐associated disc degeneration.

## 7 DIAGNOSIS OF IVDD IN DOGS

Symptoms associated with IVDD in dogs closely mimic those seen in human patients. Evidence of pain and a “hunched” or “roaching” appearance are common complaints for owners of dogs with IVDD. Other early signs include difficulty rising, getting into car, or going up stairs and/or weakness during recreational, performance, or work‐related activities or even those of daily living. These signs may be episodic, may resolve, and/or may progress to ataxia or even paralysis. For CD dogs with acute disc herniation, ataxia, or paralysis are often the first symptoms noticed by owners.

After taking a complete history and performing a general physical examination, complete neurologic examination is the foundation of diagnosis for IVDD. The goals of the neurologic examination are to localize the lesion, characterize the severity of the problem, and provide owners with an initial prognosis so that informed decision‐making regarding diagnostic imaging and treatment options can proceed. An effective neurologic examination is based on use of a comprehensive approach performed in a consistent systematic manner. The following assessments of neurologic function should be evaluated, graded, and documented using a standardized form^143^, ^144^, ^145^:

- Mentation
- Cranial nerves
- Posture, voluntary movement, and gait
- Conscious proprioception
- Spinal reflexes
- Pain perception—superficial, deep

The comprehensive, systematic neurologic examination allows the clinician to localize the lesion to forebrain, brainstem, cerebellar, vestibular, cranial nerve, peripheral nerve/neuromuscular, C1‐5, C6‐T2, T3‐L3, and L4‐S3, caudal, or multifocal. It also provides at least an initial assessment of severity of disease and prognosis. This knowledge allows the clinician and the owner to make informed decisions and directs diagnostic imaging.

After neurologic assessment and localization, diagnostic imaging is indicated to provide further detail regarding location, extent, and severity of the lesion(s) and to determine treatment options and prognosis. For dogs with spine disorders, radiographic assessment is a mainstay of diagnostic imaging in order to provide a comprehensive assessment of the patient, and radiographs alone may be sufficient for diagnosis of some disorders. When plain radiographic studies are insufficient for definitive diagnosis, advanced imaging should be performed.

Radiographic features of IVDD in dogs include narrowing of the IVD space, narrowing of the articular facet joint space, loss of the parallel orientation of adjacent endplates (“wedging”), change in shape/narrowing of the intervertebral foramen (loss of “horse head” appearance), increased opacity within the intervertebral foramen, and/or mineralized disc material within the spinal canal and/or foramen. In chronic IVDD, additional radiographic features may include proliferative changes of the articular facets, mineralization of the disc in situ, and vertebral enthesiopathy associated with spondylosis deformans (partial or bridging).^146^

Computed tomography (CT) is commonly employed for imaging cases of suspected IVDD. Multi‐slice or spiral, CT scanners are preferred when available, but single‐slice scanners can also be used effectively. Multi‐slice scanners allow for small slice thicknesses, which provide better spatial and temporal resolution, faster scan times, decrease in partial volume artifact, and decrease in motion artifacts.^147^ When using CT for spinal imaging, 1 to 2 mm slice thickness can be used with many types of scanners, which allows for accurate assessment of disc herniation location, laterality, and spinal cord compression in IVDD. Herniated material may be markedly hyperattenuating (~200 HU) to less attenuating (~60 HU), corresponding to disc material and hemorrhage, respectively. Disc degeneration with CT may be present at multiple sites with stippled mineralization at the disc space and gas present within the IVD space (vacuum effect).^147^ This herniated material in chronic disc disease can exceed 700 HU as the disc becomes more mineralized. CT myelography may be required to identify extradural compression and cord swelling in some cases.^143^, ^146^, ^148^ CT myelography features of IVDD may include soft tissue attenuating material causing compression and displacement of the spinal cord over the space of herniation and compression of the epidural space. Intravenous contrast may also be employed with a CT or CT myelogram. This may aid in the detection and differentiation of hemorrhage along the site of compression in addition to the herniated disc material.^147^

Magnetic resonance imaging (MRI) is considered to be the preferred diagnostic imaging modality for IVDD in dogs, when available. 1.5 T machines are most common in veterinary medicine with 3 T magnets becoming more commonly available at academic and large specialty centers. 3 T magnets offer improved neurologic imaging with an increase signal‐to‐noise ratio and thus improved spatial resolution. MRI spine protocols may vary depending on the manufacturer, system, and preference of the radiologist or neurologist, but a standard protocol includes sagittal, axial, and dorsal plane images in several sequences. Usually the first sequence following the localizers consists of a dorsal T2‐weighted sequence. This can then be used to accurately plan the remaining sequences and planes, which include the sagittal and transverse T2‐weighted sequence of the affected area(s) based on localization. Pre‐ and postcontrast T1 weighted images in axial and sagittal planes are performed through the area of localization. Dorsal STIR sequence may be helpful to aid in the diagnosis of paraspinal disease in some patients. Ultrafast sequences can be useful for differentiating the subarachnoid space, giving them the term “T2‐myelogram” as it has a similar appearance, allowing differentiation of extradural and extramedullary‐intradural lesions.^150^

**FIGURE 2:**
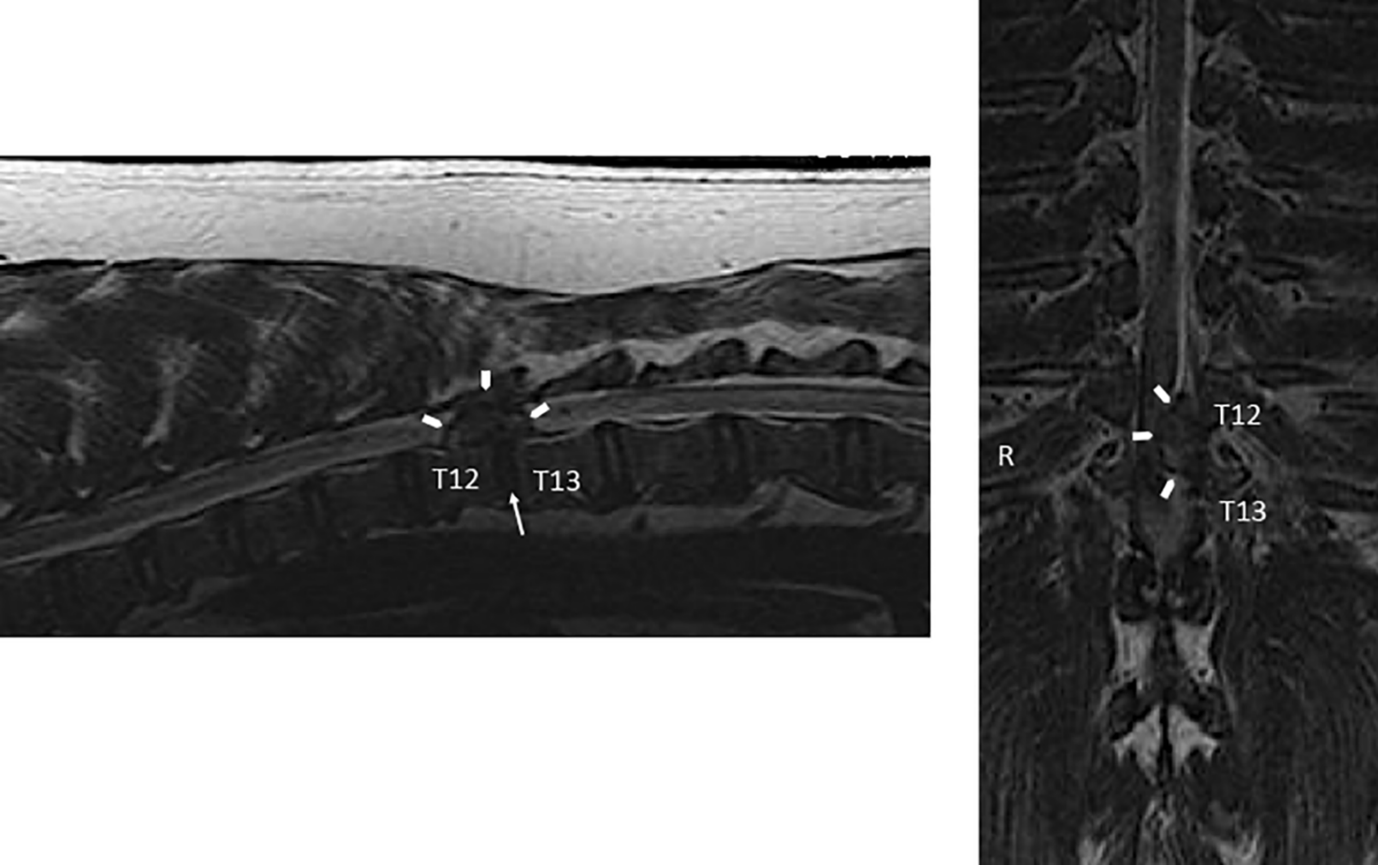
Sagittal and dorsal plane T2‐weighted MRI sequences of the thoracolumbar spine of a chondrodystrophic dog with symptomatic IVD extrusion. There is a heterogenous, extradural mass effect (arrow heads) causing compression of the spinal cord and loss of the epidural fat and subarachnoid signal at T12 to T13. There is absence of disc signal within the disc space (arrow). This is verified on the dorsal plane image with the extruded disc seen along the left side of the spinal canal, compressing the spinal cord and deviating the cord and subarachnoid signal to the right (R)

A loss of the hyperintense disc signal within the disc space on T2‐weighted sequences is associated with degeneration of the disc. Common MRI findings associated with herniation include extradural compression of the spinal cord with loss of epidural fat signal and change in spinal cord shape, change in the normal ovoid appearance of the disc, and/or narrowing of the IVD space, which is best seen on sagittal views (Figure 2). When present, epidural hemorrhage appears hyperintense to the spinal cord on T2‐weighted (T2W) images when acute or heterogenous in intensity when more chronic, depending on the magnet strength and age of the hemorrhage.^144^, ^145^, ^152^, ^153^, ^154^ Herniated disc material that compresses on the dura and spinal cord has been shown to enhance when intravenous contrast media is administered. This can occur in as much as 50% of the cases.^144^, ^155^ Myelomalacia can occur with disc extrusion and is evidenced by loss of visualization of hyperintense CSF and fat signal around the cord and diffuse to patchy hyperintensity within the cord on T2W images.^146^, ^156^, ^157^, ^158^

To the authors' knowledge, the only diagnostic imaging grading system used for canine IVD disease to date is the Pfirrmann system based on MRI. This system uses a grading scale from 1 to 5. Grade 1 is the normal, homogenous, hyperintense disc on T2 spin‐echo weighted sequences, while grade 5 is an inhomogenous, hypointense disc signal with no distinction between the nucleus and annulus and collapse of the disc space.^30^, ^159^ There was high correlation between the Thompson system of disc degeneration and the Pfirrmann scoring system using low‐field MRI in both small and large breed dogs, although there was a group of dogs that were scored higher when the presence of spondylosis was seen. Spondylosis can be seen in dogs with mild disc degeneration and even normal discs on MRI. There are several other factors that may influence the correlation of these two systems. There is variation in the size, shape, and age of the dogs; the resolution in small breed dogs is lower than in large breed dogs; and the coil effect of the MRI has brighter signal of the discs within the focus area of the MRI and decreasing signal of the disc outside of this area. This may falsely affect the grade of the disc at the periphery of the MRI focus.^30^

**FIGURE 3:**
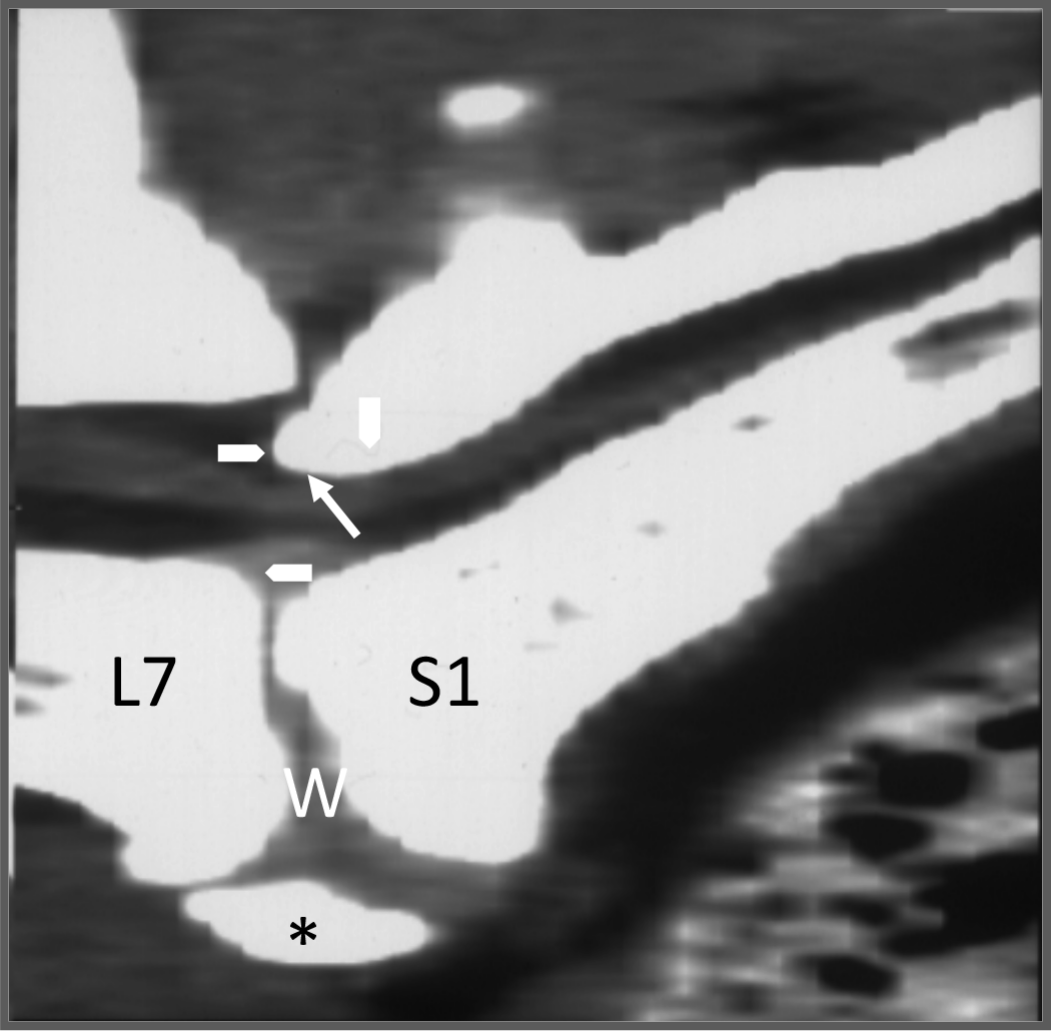
Sagittal reformatted CT image of the lumbosacral spine of a dog with lumbosacral instability and IVD disease. There is “stairstepping” (arrow heads) of the vertebral canal at the L7 to S1 junction with laminar impingement (arrow) causing deviation of the thecal sac within the canal. There is narrowing of the disc space with disc protrusion, wedging of the disc space (W), and incomplete osseous spondylosis deformans ventrally (*)

Lumbosacral disease, or cauda equina syndrome, in dogs is typically due to stenosis and/or instability and may be associated with genetic, degenerative (IVDD), overuse, traumatic, infectious/inflammatory, or neoplastic conditions. Radiographic features of LS disease may include subluxation (“stairstepping”) of L7‐S1 vertebrae, laminar and/or pedicular impingement of canal and/or foramen, disc herniation, osseous dysplasia, proliferation, and remodeling of vertebral bodies and/or articular facets, and/or sclerosis of the endplates.^146^, ^160^, ^161^ Each of these findings will be apparent on CT, which can also provide imaging data for loss of epidural fat, increased soft tissue attenuating material within the canal and foramen, thecal sac displacement, narrowed intervertebral foramen, narrowed canal, and articular facet remodeling, subluxation, and osteophytosis. Sagittal or three‐dimensional reconstruction images can be particularly useful for revealing subtler “stairstep” lesions and facet remodeling associated with LS instability (Figure 3). CT following intravenous contrast administration or CT myelography may be beneficial for ruling out trauma, infection, or neoplasia in these cases.^10^, ^146^, ^147^, ^160^, ^162^ MRI may also be additive for some cases of LS disease in dogs by providing imaging data regarding disc degeneration, disc protrusion, facet capsule and cartilage pathology, endplate, subchondral bone, and marrow lesions, and paraspinal and pelvic musculature alterations.^144^, ^146^, ^163^, ^164^ Other features that are common include spondylosis deformans, transitional lumbosacral vertebra (most commonly lumbarization of S1), swelling of the spinal nerve roots, and possible sacral osteochondrosis.^150^ The performance of “dynamic” imaging can evaluate the lumbosacral area on neutral, flexion and extension, using any of the modalities (ie, radiographic, CT, and/or MRI), to assist in the diagnosis of lumbosacral instability.^144^, ^147^, ^165^

**FIGURE 4:**
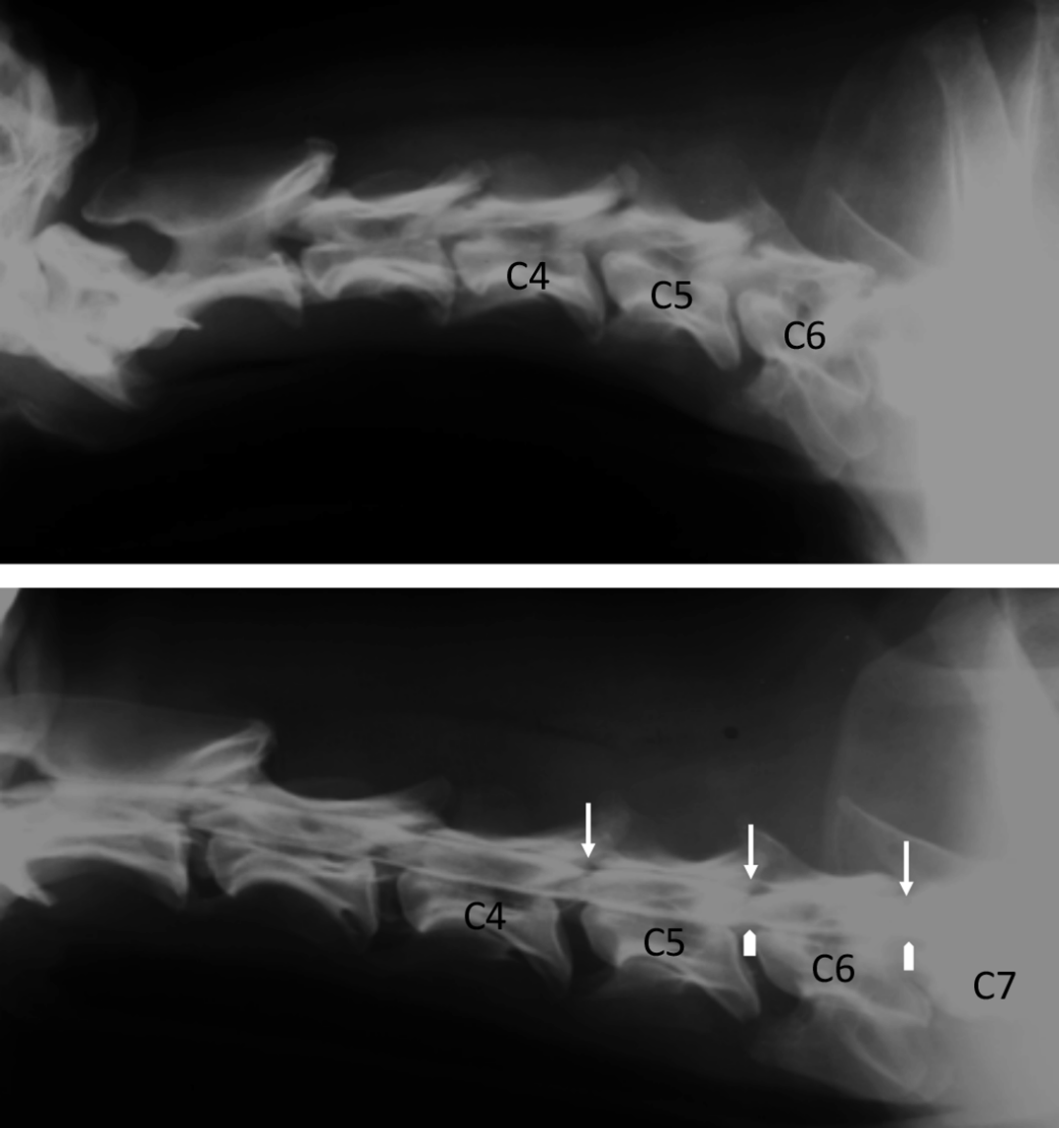
Lateral cervical spine radiographic views of a Doberman Pinscher with neurologic signs localized to the caudal cervical spine. There are subtle changes at the ventral margin of the cranial endplates at C5, C6, and C7. Lateral myelogram showing compression of the subarachnoid space/contrast column at C5 to C6 and C6 to C7 (arrow heads), and dorsal compression at C4 to C5, C5 to C6, and C6 to C7 (arrows)

In CCSM, diagnostic imaging features vary between Doberman Pinschers and Great Danes. In general, CCSM in Dobermans centers on vertebral canal stenosis, disc protrusion, and dorsal longitudinal ligament hypertrophy; whereas in Great Danes the pathology is primarily articular process malformation and hyperostosis.^146^, ^166^, ^167^, ^168^ (Figure 4) High‐quality CT images reformatted in the sagittal plane are useful for characterizing the features of CCSM. These features can include vertebral malformation, osseous protrusions into the canal (stenosis), malalignment, vertebral body malposition (craniodorsal tilting), narrowing of the IVD space, spondylosis, ligamentum flavum hypertrophy, articular process remodeling, disc degeneration, and protrusion.^13^, ^143^, ^146^, ^147^ Coronal/dorsal plane reformatted images may be helpful for the lateral compression in Great Danes with the osseous articular facet proliferation. MRI can also delineate ligamentum flavum, interarcuate ligament and joint capsule hypertrophy, synovial cysts, spinal cord compression, deformity and atrophy, AF protrusion, and NP extrusion, when present.^146^, ^169^, ^170^, ^171^ MRI of disc‐associated CCSM usually appears as an extradural, ventral compression at the disc space with the compressive lesion appearing hypointense on T1‐weighted and T2‐weighted sequences. Loss of normal disc hyperintensity can be seen at the corresponding disc space, as well as occasional hyperintensity within the spinal cord on T2‐weighted images. Foraminal stenosis is also a common feature. The osseous form of CCSM features include: hypointense T1 and T2‐weighted proliferative changes at the articular processes, lamina and pedicles, resulting in foraminal stenosis in many cases; decreased or loss of the articular process synovial fluid hyperintensity on T2‐weighted images; osseous changes resulting in vertebral canal stenosis; spinal cord compression and shape change (commonly more angular‐triangular, rectangular). Traction or kinematic (flexion/extension) MRI techniques have been described to aid in the diagnosis, localization, and severity of this syndrome.^149^

## 8 TREATMENT OF IVDD IN DOGS

Treatment of IVDD in dogs also mimics standard of care therapeutic algorithms for human patients. Symptoms of pain, stiffness, and muscle spasm without significant neurologic deficits are typically treated with oral nonsteroidal anti‐inflammatory drugs or corticosteroids (eg, prednisone), analgesics (eg, tramadol), muscle relaxants, and/or gabapentin along with activity modification and physical therapy. Corticosteroid, opioid, and/or local anesthetic epidural, sacroiliac, and facet joint injections have also been performed with success. Acupuncture and chiropractic treatments have been advocated by some, but evidence for safety and efficacy is currently lacking in veterinary medicine.

When nonsurgical treatment has failed, significant neurologic deficits are present, and/or the pathology necessitates, surgical treatment for symptomatic IVDD is indicated. The most common indication for surgical treatment of canine IVDD is acute disc extrusion in CD dogs. These cases are treated by surgical decompression and partial discectomy via partial corpectomy (“ventral slot”), laminectomy, hemilaminectomy, facetectomy, or foraminotomy of the affected disc space(s) depending on anatomic location and severity. CCSM is surgically treated by ventral (anterior) distraction‐stabilization and interbody fusion. LS stenosis and LS instability are surgically treated by laminectomy with facetectomy, partial discectomy, and/or dorsal (posterior) fusion as indicated. Traumatic spinal subluxations, luxations, and fracture‐subluxations are typically treated by open reduction and internal fixation using pins and methyl methacrylate or plates and screws, however, other methods of fixation as well as closed reduction with external fixation have also been used successfully. Importantly, adjacent segment disease is a common sequelae to IVDD and associated surgical treatments in dogs in a similar manner to that encountered in human patients.^12^, ^102^, ^172^, ^173^, ^174^, ^175^, ^176^

## 9 OUTCOME MEASURES FOR CANINE MODELS OF IVDD

### 9.1 Clinical‐functional

Based on the common and frequent management and care of IVD disorders in veterinary medicine, all of the clinical diagnostics described above can be employed in translational studies using spontaneous or induced models. Importantly, these can be performed using standard‐of‐care technology (eg, digital radiography, spiral CT, 1.5 T, or 3 T MRI) before and after treatments that are nearly identical to those performed in human patients.

For preclinical studies using canine IVDD models, inclusion of repeated neurologic exams, diagnostic imaging, and assessment of pain (CBPI^177^) is recommended. In addition, activity monitoring, kinetic, and/or kinematic assessments may be additive to specific experimental designs.^178^, ^179^, ^180^, ^181^, ^182^, ^183^, ^184^, ^185^

### 9.2 Biomechanics

The material properties of the IVD are a key measure of health and function of the spine^55^, ^91^, ^94^, ^95^, ^96^, ^97^, ^186^, ^187^ and should be included as an outcome measure when possible. The validity of any quadrupedal model of spine disease has been called into question based on the perception of fundamental differences in axial loading dynamics. However, biomechanical studies on human and canine spines have revealed that a significant amount of IVD compression can be attributed to the paraspinal musculature, suggesting that spine biomechanics are comparable between the two species.^76^, ^188^ Together with the knowledge IVDD occurs with similar frequency, mechanisms, and causes in dogs, best current evidence indicates that disc degeneration is not solely a product of human bipedal spine biomechanics^44^ and supports the use of canine models for study of the biomechanical components of IVDD as well. Ideally, the biomechanical properties of the FSUs should be evaluated in bending, compression, and rotation for pivotal preclinical studies using canine models.^96^, ^189^ Each of these tests mimics natural movements of the spine that have been validated in canine and human FSUs. These biomechanical data can then be correlated to clinical, diagnostic imaging, biomarker, macroscopic, and histologic data in order to characterize the effects of degenerative changes on function and differentiate tissue involvement, roles and mechanisms in disc health, and disease.

Incremental loading tests are designed to measure material responses to forces applied in an increasing or decreasing stepwise manner.^190^ For IVD testing, incremental loading can be applied in compression to a single disc or FSU or in compression, bending, and/or rotation to a spinal segment. Incremental loading tests are used to create force‐response profiles to characterize tissue properties.^191^ When considering biomechanical testing, the state of the sample after said testing is relevant. Compression tests are commonly performed on IVDs, FSUs, and spinal segments. As IVD compression is essentially constant due to muscle forces and gravity, and resistance to compression is a key feature of disc health, various forms of compression testing can be used to assess the compressive modulus, elasticity, creep, stress‐relaxation, and permeability of the IVD in order to characterize its functional composition, integrity, and viscoelasticity.^53^, ^64^, ^190^, ^191^, ^192^, ^193^, ^194^, ^195^ In theory, compressive, bending, rotational, biaxial, and multiaxial biomechanical tests can be incremental, single or cyclic or both, and nondestructive or destructive. If loading stays within physiologic ranges and the tissues retain their properties following testing, it can be considered nondestructive such that other assessments can be performed on the same tissues. Destructive, or load‐to‐failure, testing may be necessary based on experimental design or intended purpose of the study.

### 9.3 Histology

**FIGURE 5:**
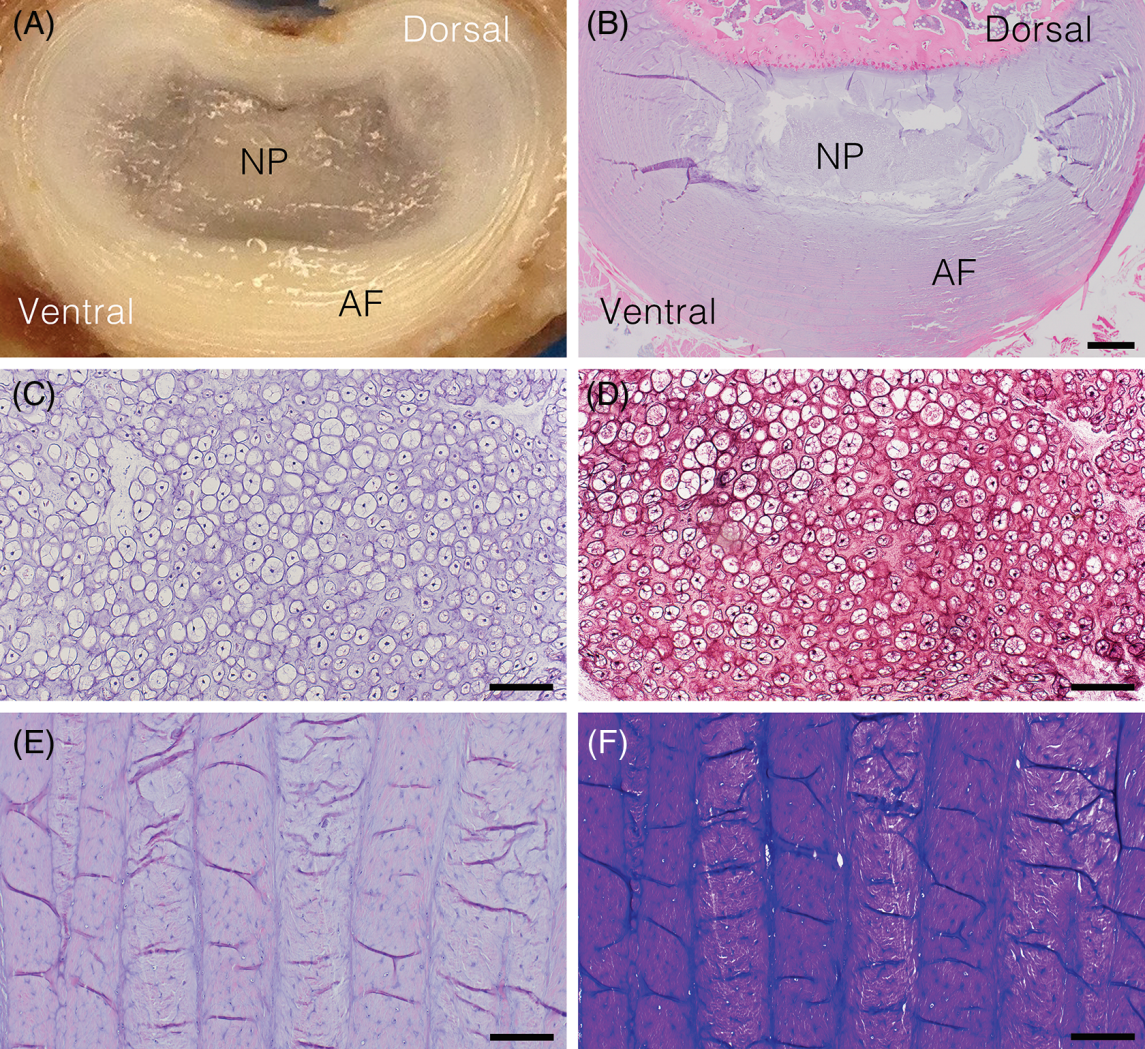
Representative images of healthy canine intervertebral discs (IVD). A, Bisected whole lumbar IVD with a gelatinous core (nucleus pulposus; NP) surrounded by rings of collagenous tissue bundles (annulus fibrosus; AF). B, Section of cervical IVD with nucleus pulposus (NP) and annulus fibrosus (AF). Scale bar = 1 mm. C,D, Higher magnification of the nucleus pulposus with large quantities of basophilic extracellular matrix populated by sheets of notochordal cells. Scale bar = 100 μm. E,F, Higher magnification of the annulus fibrosus composed of collagenous tissue bundles arranged in parallel with embedded fibrochondrocytic cells. Scale bar = 200 μm. B,C,E, H&E; D, Safranin‐O; F, Toluidine blue

Healthy IVDs are confined by two adjacent vertebrae lined by cartilaginous end plates with the interposed disc composed of a gelatinous core (NP) surrounded by rings of collagenous tissue bundles arranged in parallel (AF).^88^, ^196^, ^197^ (Figure 5) The region that interconnects AF to NP is the transitional zone (TZ).^88^ The healthy NP is composed of large quantities of basophilic ECM populated by sheets or clusters of large, irregularly‐shaped cells with a physaliferous appearance (notochordal cells).^88^, ^198^ (Figure 5) The TZ contains chondrocyte‐like cells embedded in a loose, collagenous tissue.^88^ Fibrochondrocytic cells are embedded within the normal AF; the cells of the outer AF are predominantly fibroblasts/fibrocytes, while the cells of the inner AF are a mixture of fibroblasts/fibrocytes and chondrocyte‐like cells.^88^ The cartilaginous endplates resemble hyaline cartilage.^38^ The cartilaginous endplates are thicker in human IVDs compared to canine IVDs.^45^ This difference is thought to exist because vertebral growth in dogs is primarily regulated by separate epiphyseal growth plates located at both the cranial and caudal ends within the vertebrae, while in humans the vertebral growth mainly occurs in the interface between the cartilaginous endplates and the subchondral bone.^45^

**FIGURE 6:**
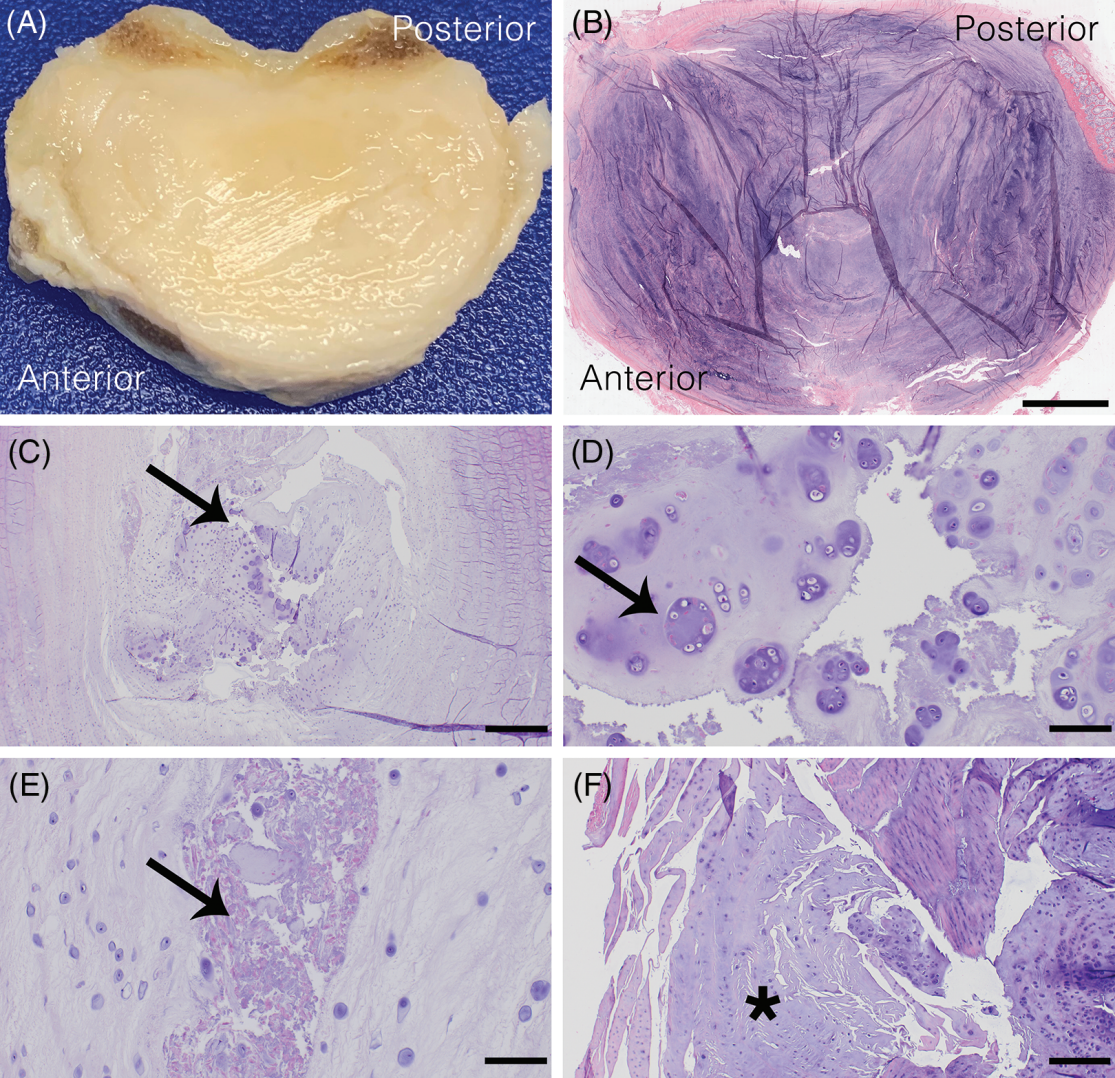
Representative images of degenerative changes in human and canine intervertebral discs (IVD). A, Bisected whole human thoracic IVD from a 43‐year old male. B, Section of human lumbar IVD from the same male. The annulus fibrosus‐nucleus pulposus demarcation is completely lost. Scale bar = 1 cm. C, Tissue fissure/cleft formations (arrow) in the canine nucleus pulposus with loss of notochordal cells and cellular proliferations. Scale bar = 1 mm. D, Loss of notochordal cells and proliferation of chondrocytic‐like cells and clone formation (cell aggregates; arrow) in the canine nucleus pulposus. Scale bar = 100 μm. E, Granular debris (arrow) at the transitional zone of canine IVD. Scale bar = 100 μm. F, Loss of collagenous meshwork and replacement by increasingly hyalinized collagen fibers (*) in the canine annulus fibrosus. Scale bar = 500 μm. B‐F, H&E

Grossly, degenerative NP is more solid (less gelatinous) and opaque, tears are often visible in the AF and/or NP, the AF‐NP demarcation is lost, and there are irregularities in the endplates.^199^, ^200^ (Figure 6) Best current evidence suggests that the cellular changes observed during the course of IVDD in CD and NCD dogs are both consistent with chondroid degeneration^31^, ^201^ and similar to the degenerative processes noted in human IVDD.^45^ The only major differences noted among these cohorts are related to the timing of notochordal cell senescence. In humans, notochordal cells are senescent in the fetus and disappear within the first 11 to 16 years of life.^202^ CD dogs show premature senescence of notochordal cells with replacement by chondrocyte‐like cells (chondroid metaplasia) usually within the first year of life (more similar to humans), while NCD dogs maintain a more mucoid NP with a delay of notochordal cell senescence/chondroid metaplasia into adulthood (5‐7 years of age).^45^

Histologic features of disc degeneration include tissue fissures/clefts, mucoid degeneration, loss of notochordal cells in NP, proliferation of chondrocytic‐like cells and clone formation (cell aggregates), cell death, loss of AF‐NP demarcation, granular debris, fibrosis, and neovascularization from the outer AF inward.^31^, ^88^, ^197^, ^202^ (Figure 6) Decreasing proteoglycan content of NP is associated with an increased severity of IVD degeneration.^88^ Gradual loss of collagenous meshwork and replacement by increasingly hyalinized collagen fibers is observed in degenerative AF.^202^ (Figure 6) Changes in the cartilaginous endplate and neighboring bone include cell proliferation of chondrocytes, cartilage disorganization (irregular thickness) and defects, new bone formation, and subchondral bone sclerosis.^202^ In humans, spondylosis is generally associated with advanced IVD degeneration, while spondylosis in dogs is relatively common and can be associated with healthy or mildly degenerative IVDs.^200^

For pivotal preclinical translational studies using canine models, histologic assessments at a relevant endpoint are typically recommended and may be required for regulatory approval of pharmaceuticals, biologics, devices, or other interventions. Comprehensive assessments using applicable staining techniques and validated grading/scoring systems should be employed. Hematoxylin & eosin staining characterizes the tissue architecture and cellular changes of the whole IVD. Toluidine blue, Safranin‐O, or Alcian blue determines polysaccharide content and distribution within NP and AF.^88^, ^203^ Picrosirius red stains collagens. A novel staining procedure using Alcian blue and Picrosirius red (ABPR) can assess changes in intercellular and ECM components based on distinctive staining of collagen (red) and proteoglycans (blue).^202^, ^203^, ^204^, ^205^ Immunohistochemistry is not routinely used to assess degenerative changes in IVDs; however, it can be used to localize and identify collagen and matrix components. The Thompson grading scheme for degenerative changes in human IVDs quantifies the gross morphology of midsagittal sections of human lumbar IVDs^199^ and this scoring system has been validated for use in dogs.^200^ It is a five‐category scheme (ie, I, II, III, IV, or V) for assessing pathological changes of the NP, AF, endplates, and periphery of the vertebral body.^199^ Grade I is considered normal (juvenile disc), while grade V represents end‐stage degeneration.^199^ For humans, the Boos et al histological grading scheme^202^ is the most commonly used validated classification system for midsagittal sections of human IVDs. The IVD (NP and AF) is graded on five different categories: chondrocyte proliferation, mucous degeneration, cell death, tear and cleft formation, and granular changes.^202^ There are six different items graded for the endplate: cell proliferation, cartilage disorganization, cartilage cracks, microfracture, new bone formations, and bony sclerosis.^202^ A new validated histological classification for human IVDs has been developed by Rutges et al with only six different categories and three different scoring options per category.^203^ This classification system is less complex than the Boos et al grading scheme with 11 categories and between three and seven different scoring options per category. The Bergknut et al histological grading scheme is commonly used to classify degenerative changes in both CD and NCD dog breeds.^205^ Nine separate variables evaluate cytological changes, alterations in matrix composition, and structural changes seen in degenerative IVDs: the morphology of AF, chondrocyte metaplasia of AF, tears and cleft formation, chondrocyte proliferation of NP, presence of notochordal cells in NP, matrix staining of the NP, endplate morphology, new bone formation, and subchondral bone sclerosis.^205^ This grading scheme was initially developed for the post‐mortem evaluation of whole IVDs^205^; however, a recent study indicated that this scheme could also be used for canine surgical IVD biopsies by omitting variables not visible in the biopsies.^201^ For tears and cleft formations, it is important to distinguish between artifacts caused by shrinkage and dehydration during tissue processing (not associated with cellular/matrix change) and “true” tears/clefts commonly associated with other degenerative changes, such as chondrocyte‐like cell proliferation and mucous degeneration.

Pathological changes are often not uniformly distributed throughout the IVD, so discrepancies between gross and histological assessments can occur. Localized, severely degenerated parts of the IVD might not be grossly visible, yet can disproportionately skew histologic grades and scores if preferentially sectioned for microscopic evaluations.^31^, ^205^ As such, comprehensive whole‐organ grading and scoring methods are recommended, and macroscopic and microscopic pathology data should be compared with and correlated to clinical, diagnostic imaging, biomarker, and biomechanical data in order to appropriately characterize the severity and impact of degenerative changes on mechanisms and clinical applications in the study of IVDD.

### 9.4 Biomarkers

The National Institutes of Health's Biomarker Definitions Working Group defines a biomarker as “a characteristic that can be measured and evaluated as an indicator of normal biologic processes, pathologic processes or pharmacologic responses to therapeutic intervention.”^57^ Currently, there are no biomarkers that have been validated for clinical use to diagnose, stage, prognosticate, or assess outcomes for any component of IVDD in dogs or humans. However, intensive research in this arena is being pursued using in vitro, translational, and clinical studies, and preclinical canine models provide a powerful tool in this effort.

The ideal biomarker(s) for IVDD would provide precise, accurate, and early information for diagnosing and categorizing likelihood, type and severity of disease, for deciding timing and type of intervention, for evaluating response to treatment, and for determining prognosis using an easy‐to‐obtain and minimally invasive sample, such as oral swabs, blood, or urine. For example, a panel of molecular biomarkers from an oral swab for polymerase chain reaction analyses could determine relative risk for symptomatic and progressive scoliosis in pediatric patients, or a panel of serum protein biomarkers could determine likelihood for response to nonsurgical management of low‐back pain in men over 60 years of age. For each of these examples, the appropriate intervention could then be determined and implemented with higher likelihood for compliance and success. Similarly, veterinarians could use a panel of urine protein biomarkers to monitor Dachshunds at annual wellness appointments for progression of disc degeneration associated with extrusion, or they could use a panel of molecular biomarkers from puppies bred for military service to ascertain relative risk for lumbosacral instability prior to assignment to time‐ and cost‐intensive training. Both of these veterinary medical examples would also effectively inform breeding decisions.

In humans and dogs, IVDD is associated with inflammation, altered matrix synthesis, catabolic metabolism, cell death, and neural and vascular ingrowth in the disc and surrounding tissues.^206^ As such, MMPs, ADAMTs, cytokines, chemokines, and ECM proteins have been the main focus of biomarker studies.^207^, ^208^, ^209^ Proteomics^198^, ^210^, ^211^, ^212^, ^213^, ^214^, ^215^ and metabolomics^216^ are the tools often used to identify and develop biomarkers of IVDD. In dogs, 15F_2t_ isoprostane in urine of IVDD patients,^217^ Runx2 Runt‐related transcription factor 2 (Runx2) expression in calcified IVDs in Beagle dogs,^218^ NG2 proteoglycan expression in degenerative Dachshund IVDs,^219^ and Link‐N that interacts with proteoglycan aggregates^220^ in CD and NCD dogs have been reported as having potential to serve as clinically relevant biomarkers. There are other biomarker studies in dogs that focus on molecular signatures of inflammation such as IL‐6, IL‐8, and other cytokines and chemokines in degenerative IVDs.^44^, ^208^, ^221^

Based on the breadth of similarities between human and canine IVDD and the currently unmet need for clinically relevant biomarkers in human and veterinary medicine, the authors have pursued programmatic research aimed at identifying protein biomarker panels for spine disorders. To pursue this aim, ongoing basic science, translational, and clinical studies have been designed to develop and validate IVDD biomarker panels from serum, urine, tissue, and culture media from CD and NCD dogs with or without symptomatic IVDD, human patients being treated for spine pain and those requiring surgical treatment for IVDD, and organ and tissue donors (ORS PSRS 2019, ORS 2020 poster#1879, 0978, 0979, and 1861). Briefly, initial results suggest that canine CD and NCD and human IVDs respond to IL‐1β stimulation by increasing IL‐6, MCP‐1, and CXCL1 production. Potentially differentiating biomarkers in these studies include IL‐8 and MMP‐1,2, and 3, which suggest that CD dogs may best model more acute IVD disorders in younger patients, while NCD dogs may best model more chronic IVD degeneration in older patients. Further characterization of biomarker production patterns will help delineate potential clinical applications for both species. If conserved protein expression or metabolite signatures of IVDD can be identified in both species, the fundamental process that lead to early degeneration of IVDs could be explored as diagnostic, prognostic, preventative, and therapeutic targets.

## 10 CONCLUSIONS

Dogs provide powerful models for disorders of the spine. Pathogenesis, causes, clinical presentations, and diagnostic tools for IVDD are highly similar between human and canine patients. In particular, spontaneously occurring IVD degeneration in CD and NCD breeds of dogs provide highly translatable preclinical data for symptomatic disc degeneration disorders seen across the spectrum of age‐, cause‐, and pathology‐associated patient cohorts. Measurable data obtained through scientific studies in dogs provide insights into histopathology, biomechanics, and various biomarkers with high clinical relevance, but that cannot be practically or ethically obtained from human patients.

When choosing a preclinical model for spine research, it is critical to remember that biologic and biomechanical components of IVD health and disease are inextricably linked. Furthermore, it is important to acknowledge and address limitations of canine models including subtle differences in vertebral column anatomy, biomechanics, genetics, physiology, and lifestyles. As such, comprehensive outcome assessments with correlations among metrics are important for validity and translatability. The spontaneously‐occurring canine models described in the present review article are amenable to this comprehensive and correlative approach while also corresponding directly to in vitro, ex vivo, and induced‐disease canine models. Taken together, preclinical studies using the full breadth of canine models can guide targeted research toward developing valid and effective tools for early diagnosis, prevention, and treatments both for human and canine patients.

## CONFLICT OF INTEREST

The following authors have the following declarations: Naomi N. Lee: No conflicts to declare; Jacob S. Kramer: No conflicts to declare; Aaron M. Stoker: Arthrex, Inc: IP royalties; Other financial or material support; Musculoskeletal Transplant Foundation: IP royalties; Chantelle C. Bozynski: No conflicts to declare; Cristi R. Cook: Arthrex, Inc: IP royalties; Paid consultant; Paid presenter or speaker; Research support CONMED Linvatec: IP royalties; Paid consultant; Paid presenter or speaker Musculoskeletal Transplant Foundation: IP royalties; Paid presenter or speaker Zimmer: Research support; James T. Stannard: No conflicts to declare; Theodore J. Choma: AO Spine North America: Board or committee member Gentis, Inc: Stock or stock Options North American Spine Society: Board or committee member Scoliosis Research Society: Board or committee member; James L. Cook: Artelon: Paid consultant Arthrex, Inc: IP royalties; Paid consultant; Paid presenter or speaker; Research support AthleteIQ: IP royalties ConforMIS: Research support CONMED Linvatec: Paid consultant Coulter Foundation: Research support DePuy Synthes, A Johnson & Johnson Company: Research support Eli Lilly: Paid consultant; Research support Journal of Knee Surgery: Editorial or governing board Merial: Research support Midwest Transplant Network: Board or committee member Musculoskeletal Transplant Foundation: Board or committee member; IP royalties; Research support National Institutes of Health (NIAMS & NICHD): Research support Purina: Research support Schwartz Biomedical: Paid consultant Thieme: Publishing royalties, financial or material support Trupanion: Paid consultant U.S. Department of Defense: Research support Zimmer‐Biomet: Research support. *Note*: Authors James L. Cook and Cristi R. Cook are husband and wife.

## AUTHOR CONTRIBUTIONS

All authors have read and approved the final submitted manuscript. The following is the author contribution: Naomi N. Lee, James L. Cook: substantial contributions to research design, acquisition, analysis, and interpretation of current literature; Naomi N. Lee, Jacob S. Kramer, Aaron M. Stoker, Chantelle C. Bozynski, Cristi R. Cook, James T. Stannard, Theodore J. Choma, James L. Cook: drafting the paper and revising it critically; Naomi N. Lee, Jacob S. Kramer, Aaron M. Stoker, Chantelle C. Bozynski, Cristi R. Cook, James T. Stannard, Theodore J. Choma, James L. Cook: approval of the submitted and final versions.
